# Wild-type transthyretin amyloidosis in female patients

**DOI:** 10.1186/1750-1172-10-S1-O9

**Published:** 2015-11-02

**Authors:** Arnt V Kristen, Ralf Bauer, Fabian aus dem Siepen, Christoph Kimmich, Katrin Hinderhofer, Christoph Röcken, Hugo A Katus

**Affiliations:** 1 Department of Cardiology, University of Heidelberg, 69120, Heidelberg, Germany; 2 Department of Hematology, University of Heidelberg, 69120, Heidelberg, Germany; 3 Institute of Human Genetics, University of Heidelberg, 69120, Heidelberg, Germany; 4 Institute of Pathology, University of Kiel, 24105, Kiel, Germany

## Background

Wild-type TTR amyloidosis (wt-ATTR) is a common aging phenomenon in the elderly population. It is claimed to affect exclusively males. Female gender was assumed as a protective factor. Clinical data on wt-ATTR in female gender is lacking. This single center analysis reported on gender differences of clinical variables in wt-ATTR.

## Methods

Patient records of 207 consecutive patients with wt-ATTR were analyzed for clinical variables obtained during the initial assessment at Heidelberg Amyloidosis Center, including electrocardiography, echocardiography, and laboratory results. All variables were compared between male and female gender. Finally, predictors of survival (onset of first symptoms to death) were evaluated.

## Results

Comparison of clinical findings between males and females affected by wt-ATTR amyloidosis are shown in table 1. Female patients with wt-ATTR did not differ from male patients regarding demographic or clinical parameters except for modified body mass index (1140±184 vs. 1029±154, p<0.05), glomerular filtration rate (66±23 vs. 85±31 ml/min*m^2^*1.73; p<0.05), NYHA class (2.4±0.7 vs. 2.9±0.3; p<0.01) and PQ interval (211±50 ms vs. 170±26; p<0.01). Interestingly, both groups especially did not differ in age at onset of symptoms, but longer delay between start of symptoms and diagnosis of wt-ATTR in females was observed when compared to male patients with wt-ATTR.

In total, 6 deaths (35%) occurred in females and 45 deaths (24%) in males. No gender differences were observed regarding mean survival (females 54±35 month, males 56±107 months). By multivariate analysis independent predictors of mortality in the whole cohort were use of diuretics (HR 8.657, 95%CI 1.160-64.17; p=0.035) and hs-TnT (HR 1.009, 95%CI 1.004-1.015; p=0.001).

In total, 6 deaths (35%) occurred in females and 45 deaths (24%) in males. No gender differences were observed regarding mean survival (females 54±35 month, males 56±107 months). By multivariate analysis independent predictors of mortality in the whole cohort were use of diuretics (HR 8.657, 95%CI 1.160-64.17; p=0.035) and hs-TnT (HR 1.009, 95%CI 1.004-1.015; p=0.001).

## Conclusion

Although wt-ATTR is claimed to be a disease of male gender there is a considerable number of females affected with cardiac manifestation of wt-ATTR. According to this first report on clinical characteristics of a relatively well sized cohort of females no gender-specific differences regarding clinical characteristics and median survival were observed, except for modified body mass index and PQ interval as well as higher glomerular filtration rate and NYHA class in female patients. Moreover, use of diuretics and hs-TnT appeared to be predictors of mortality.

